# Construction and assessment of the “Almanac for Women with Breast Cancer”: convergent care research

**DOI:** 10.1590/0034-7167-2025-0031

**Published:** 2025-12-12

**Authors:** Karolina Rosa Teófilo, Denise Antunes de Azambuja Zocche, Ana Paula Carmona, Bruno Henrique Fiorin, Marcia Valéria de Souza Almeida, Cândida Caniçali Primo, Eliane de Fatima Almeida Lima

**Affiliations:** I Universidade Federal do Espírito Santo. Vitória, Espírito Santo, Brazil; II Universidade do Estado de Santa Catarina. Florianópolis, Santa Catarina, Brazil; III Escola Superior de Enfermagem de Lisboa. Lisboa, Portugal

**Keywords:** Breast Neoplasms, Health Education, Educational Technology, Nursing Care, Mastectomy., Neoplasias de Mama, Educación Sanitaria, Tecnología Educativa, Atención de Enfermería, Mastectomía.

## Abstract

**Objectives:**

to develop a printed almanac for women with breast cancer.

**Methods:**

convergent care research with a qualitative approach, carried out in a specialized oncology service in Vitória, Espírito Santo, from August 2023 to August 2024. The almanac was built based on the doubts of women undergoing chemotherapy treatment. After its creation, participants used and assessed the technology. An agreement rate of 80% was considered adequate.

**Results:**

the almanac has 47 pages, with comic strips and games such as word searches, seven differences, mazes and others. Participants assessed the educational technology as relevant, enlightening and capable of encouraging new habits, in addition to adding knowledge. All items had an agreement rate above 80%.

**Conclusions:**

the almanac consists of an innovative educational technology that addresses risk factors, treatments, self-care and social rights of people with cancer in a playful way and with easy-to-understand language.

## RESUMEN

**Objetivos:**

desarrollar un almanaque impreso para mujeres con cáncer de mama.

**Métodos:**

investigación de atención convergente con enfoque cualitativo, realizada en un servicio de oncología especializado en Vitória, Espírito Santo, entre agosto de 2023 y agosto de 2024. El almanaque se elaboró a partir de las dudas de mujeres en tratamiento de quimioterapia. Tras su creación, las participantes utilizaron y evaluaron la tecnología. Se consideró adecuado un índice de concordancia del 80%.

**Resultados:**

el almanaque cuenta con 47 páginas, con tiras cómicas y juegos como sopas de letras, siete diferencias, laberintos y otros. Los participantes evaluaron la tecnología educativa como relevante, enriquecedora y capaz de fomentar nuevos hábitos, además de enriquecer el conocimiento. Todos los ítems tuvieron un índice de concordancia superior al 80%.

**Conclusiones:**

el almanaque consiste en una tecnología educativa innovadora que aborda los factores de riesgo, los tratamientos, el autocuidado y los derechos sociales de las personas con cáncer de forma lúdica y con un lenguaje fácil de entender.

## INTRODUCTION

Breast cancer remains the most common cancer and the leading cause of death among women worldwide. In 2022, according to estimates from the Global Cancer Observatory, of the 19.9 million new cases of cancer diagnosed worldwide, 23.8% were related to breast neoplasms. In Brazil, it is estimated that, in the three-year period 2023-2025, there will be 74 thousand new cases of the disease, with 66.54 cases/100 thousand women. It is worth noting that breast cancer is the leading cause of death, with 16.47 deaths/100 thousand women, in 2020^(1,2)^.

The different treatment modalities for breast cancer expose women to several undesirable effects that must be assessed by the multidisciplinary team for therapeutic planning that aims to offer health information, comfort and self-care promotion^(3,4)^.

Health education actions are one of the fundamental pillars for health promotion and prevention, expanding people’s health literacy. Educational materials on breast cancer should provide clear and accurate information to patients^(5,6)^.

Many women around the world still have limited knowledge about the symptoms and risk factors of breast cancer. In view of this, it is essential that healthcare professionals improve health education actions aimed at preventing this type of cancer, promoting awareness of its importance^(7)^. Educational technologies assist in this process, as they can expand the knowledge of women diagnosed with the disease and improve their quality of life, allowing patients to take a leading role in their treatment^(8)^.

In this context, different technological and educational resources can be used to carry out educational interventions on breast cancer, which are effective and low-cost strategies to be directed at men and women at all stages of life^(9)^.

Among the various educational technologies, almanacs stand out for being informative, humorous, playful and dynamic. They facilitate the transition from oral to written culture, presenting content in a recreational and systematic way. Moreover, they use simple and easy-to-understand language, making information more accessible to everyone^(10-12)^.

Thus, considering that cancer treatment, whether systemic, such as chemotherapy, or local, such as radiotherapy and surgery, can cause psychological, emotional, social and physical side effects, the different treatment modalities should be addressed by the health team in order to reduce the doubts and fears of women diagnosed^(7)^. Thus, a study was developed to develop educational material for women diagnosed with breast cancer, in order to clarify their doubts about treatment as well as to provide support for use by healthcare professionals during verbal guidance. In this sense, the target audience’s participation in this development was essential to meet their real needs.

## OBJECTIVES

To develop a printed almanac for women with breast cancer.

## METHOD

### Ethical aspects

The research project was approved by the Research Ethics Committee, under Certificate of Presentation for Ethical Consideration 70587623.6.0000.5060.

Participants were informed about the objective and development stages of the research. After reading the Informed Consent Form, participants signed and received a copy of the form. The interviews were conducted in a private location, and participant anonymity was preserved by using the letter “P” to identify each interviewee. Participants were informed that they could leave the research at any time and that there would be no implications for continuity of their care.

### Theoretical-methodological framework

The study used the convergent care research (CCR) method, which consists of the articulation between research actions and care practice, allowing the acquisition of information in both processes^(13)^.

### Study setting

The study was carried out in a specialized oncology service in the city of Vitória, Espírito Santo, from August 2023 to August 2024.

The research followed the steps described by the CCR methodological model, which consists of design, instrumentation, investigation and analysis^(13)^.

In the design phase, the research questions were defined based on the researcher’s care practice, who took on a proactive role, seeking convergence between research and care actions. Also in this phase, a narrative bibliographic review was carried out on the topic in order to bring the researcher’s knowledge closer to the evidence found in the literature. Based on these results, the research proposal was presented to the institution’s nursing team before starting the data collection phase, in order to involve them in the study so that any problems that arose from the data obtained could be shared with the purpose of promoting discussion and problem-solving.

In the instrumentation stage, methodological issues were defined, such as the research setting, participant characterization and the data collection technique.

Participants were women with breast cancer undergoing chemotherapy, selected by convenience based on their involvement with the subject, according to their attendance at the location for treatment.

Women diagnosed with breast cancer undergoing treatment and who were literate were included. Women who underwent curative surgery as the only treatment (this procedure is performed in an external service) and those who had a personal history of psychiatric or cognitive illness were excluded.

Data collection occurred through individual semi-structured interviews conducted during participants’ infusion treatment. At first, the research was presented, the Informed Consent Form was read and, after it was signed, the interview was conducted. There were no refusals. To guide the research, the COnsolidated criteria for REporting Qualitative research were used^(14)^.

The 16 interviews took place from October 8, 2023 to February 6, 2024; they were recorded on MP3 and lasted approximately 30 minutes. The interviews ended when theoretical data saturation occurred, with no new content emerging.

During the interview, a form structured by the author was used to identify sociodemographic and economic data, and the type of educational technology chosen by participants (almanac, gamebook, comic book and word search). Also, during the interview, participants were asked about what guidance they received from healthcare professionals after diagnosis, their knowledge about self-care and the social rights of people with cancer, about what treatments they underwent, and whether they were advised about possible side effects.

The recordings of the interviews were transcribed in full by the author and read exhaustively. For the categorical content analysis, as described by Bardin, the following phases were followed: pre-analysis, material exploration, and treatment of results, inference and interpretation^(15)^.

In the investigation phase, the educational technology was developed in the format of an almanac (type chosen by the participants), including content suggested based on the questions presented by them or topics that they considered important to compose the material.

For the theoretical basis, a narrative bibliographic review was carried out on the main topics identified in the interviews. Contents were sought in the Ministry of Health manuals, booklets related to breast cancer, scientific articles published in the Virtual Health Library, in the Medical Literature Analysis and Retrieval System Online databases, Latin American and Caribbean Literature in Health Sciences and Nursing Database^(16-24)^. To select educational games, a survey was carried out in existing pastime magazines, primers and almanacs.

The data analysis and interpretation phase supported the process of developing the almanac, following the phases of organizing the content based on scientific research, developing the pilot model, and illustrating and laying out the almanac. In this regard, the almanac prototype was developed based on photos taken by the researcher, representing the various treatment scenarios for women with breast cancer, in order to express the idea proposed by the educational technology. The prototype content was corrected in terms of illustration, layout and language, with the collaboration of a team consisting of a designer, a literature professional and a healthcare professional.

In the assessment stage, the same participants received the almanac, and after a week of using the material, they were interviewed again. The interviews took place from July to August 2024. In the interview, the adapted form based on the instrument for validating educational content in health with material objectives, structure/presentation and relevance^(25)^ was applied, and they could request clarification if they deemed it necessary. They marked with an “x”, according to their judgments, the following dichotomous variables: 1 - agree; and 2 - do not agree. The items that reached a minimum agreement of 80% were not changed, as they were considered adequate. During the interview, participants were asked about how it was for them to read and use the almanac, what their perceptions of the material were, the positive points and what could be improved.

## RESULTS

Sixteen women diagnosed with breast cancer and undergoing intravenous drug treatment, with an average age of 46.9 years, participated in the study. Of these, 62.5% had completed higher education; 93.75% reported having a steady job; and 81.2% were married. Concerning the number of children, 75% of women had between one and two children and lived in municipalities in the greater Vitória area, Espírito Santo, Brazil.

The educational technology developed, entitled “Almanac for Women with Breast Cancer”, consists of a printed material with 47 color pages, produced on coated paper, measuring 21 cm in height by 15 cm in width. The content is presented through stories and games starring characters Ana (patient), Zeca (husband), Rafa (son), grandma Lena, and nurse Flora. Part of this material is described throughout this article.

**Figure 1:**
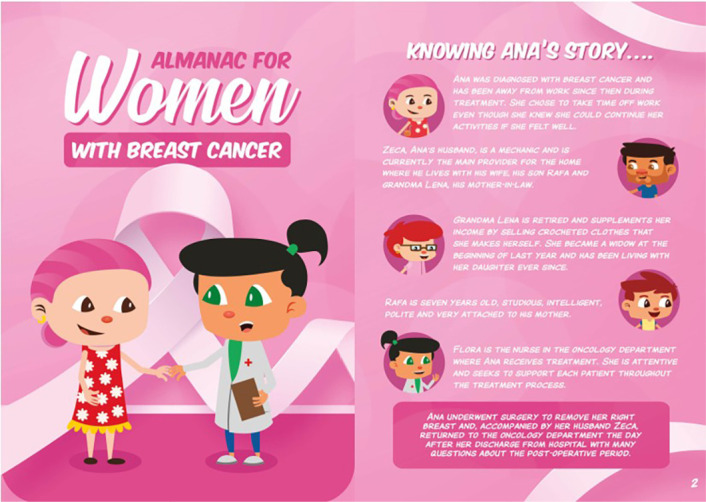
Almanac cover and character presentation, Vitória, Espírito Santo, Brazil, 2024

The almanac cover is attractive and seeks to convey accessibility, partnership, and security between women and healthcare professionals (Figure 1). Its purpose is to reduce the distance reported by participants, caused by fragmented and incomplete guidance during treatment.

Eight comic strips were created with topics chosen by participants, such as mastectomy, post-operative, risk factors, social rights, chemotherapy, radiotherapy, leisure and completion of treatment. To stimulate learning, the almanac includes tips from the nurse, recipes and 11 pastime magazines, such as word searches and crosswords. It also features a calendar for treatment planning.

In relation to content related to surgeries (mastectomy and quadrantectomy), care with the drain stands out, since, of the 13 participants who underwent some surgical procedure, nine used the device in the post-operative period and only six received guidance, but in an inadequate manner, with incomplete information and without scientific basis, as demonstrated in:

*Regarding the drain, I think the care was to apply a little medicine, Rifocin* [...] *like Merthiolate, I used it a lot.* (P8)

*They told my husband, “When the drain reservoir is full, you go to the toilet, open this here and turn it over and close it”. But one day I was sleeping and the reservoir came loose, I told my husband, “Wash the thing* [drain] *now and put it back in”.* (P16)

**Figure 2:**
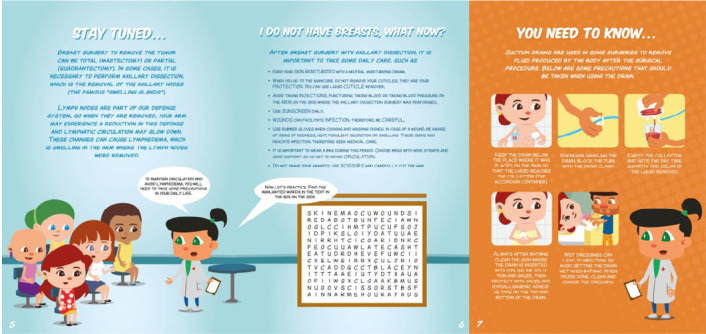
Information about mastectomy and post-surgery care in the almanac, Vitória, Espírito Santo, Brazil, 2024

To guide women about mastectomy and post-procedure care, games and tips on the subject were developed (Figure 2).

Participants also raised questions related to risk factors for the development of breast cancer, recurrences and the HER2+ type of cancer, as explained in the statements:

*I don’t remember the risk factors for developing breast cancer.* (P12)

*In reality, I always have some doubts.* [...] *I have a recurrence, so I keep thinking “what can I do to prevent this from happening again?”. Did I make a mistake? Could I have done something? Why did this happen?* (P14)

**Figure 3:**
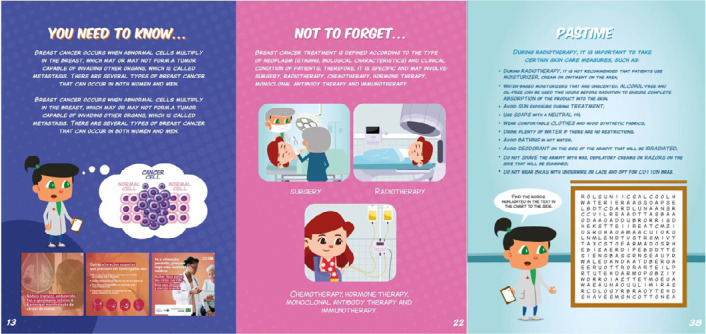
Information about cancer development, treatments and care during radiotherapy, Vitória, Espírito Santo, Brazil, 2024

Chemotherapy and radiotherapy were presented through comic strips contextualizing the treatment as well as precautions for preventing adverse reactions and the use of cryotherapy. Games and tips on the subject were also developed in order to convey the knowledge in a simple, playful and easy-to-understand manner (Figure 3).

**Figure 4:**
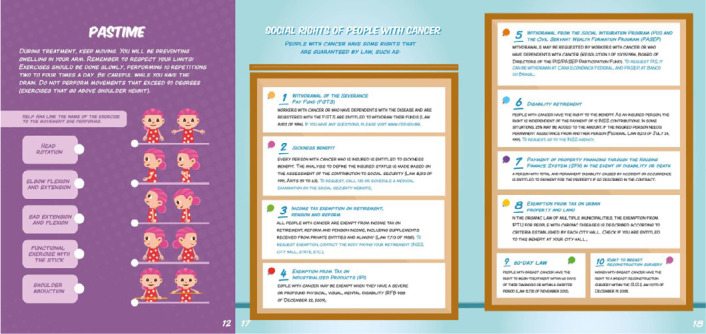
Pages from the almanac with information on exercises for preventing lymphedema and tables describing some social rights of people with cancer, Vitória, Espírito Santo, Brazil, 2024

The almanac included suggestions for physical exercises to prevent lymphedema and some social rights guaranteed by law for people diagnosed with cancer, such as sickness benefit, income tax exemption, disability retirement and others (Figure 4).

*I don’t know what lymphedema is.* [...] *the doctor just told me not to put pressure on the arm on the side where I had surgery.* (P8)

*At the moment, I don’t know any social rights I have because I’m undergoing treatment, but I would like to know.* (P10)

The almanac ends with the story “Ana in: finalizing treatment”, in which Ana and Zeca return for a consultation with nurse Flora, who provides guidance on follow-up after the end of treatment.

The answer key for the games was included on the last page of the “Almanac for Women with Breast Cancer”.

The information followed a chronological order that may differ, since treatment is defined according to the characteristics of each disease and the clinical condition of each person.

In the almanac assessment phase, six participants were lost due to death, communication difficulties for the delivery of the educational technology and for scheduling the interview, and disease progression, which made it impossible to read. Thus, ten women responded to the assessment form.

In the technology assessment, all items achieved agreement above 80%; therefore, they were considered adequate.

Most participants (90%) reported that the content is informative, encourages new habits and addresses important issues that most women have questions about.

*Everything there is great for me, it cleared up a lot of my doubts, especially the issue of social rights.* [...] *there are a lot of things there that I didn’t know.* (P1)

*It encourages new eating habits and the importance of physical activity.* (P3)

*So, I wish I had an almanac like that when I was diagnosed. I think it addresses the information very clearly.* (P7)

On the other hand, 10% disagreed that the almanac covered all proposed topics, as it did not cover in depth the types of breast cancer such as HER2 positive or negative, triple negative, etc. However, these items were not included because this information should be provided by healthcare professionals in line with the protocol that will be followed, with the almanac being a support technology for the health education process.

Concerning structure/presentation, most participants considered the material to be objective and enlightening, in addition to the importance of including the support network:

*The material is light, uncomplicated and informative. It addresses important issues that the vast majority of patients have doubts about.* (P2)

*I liked it, I liked it a lot, I thought it was great, very explanatory.* [...] *I found the games creative and fun.* (P9)

*I thought it was interesting to include the family during the treatment.* [...] *the colors and the printing material are conducive to reading.* (P13)

One participant found the language in the comic strips childish and suggested changing “measure blood pressure” to “check blood pressure” (page 04). To expand the vocabulary, both terms were kept, with one in parentheses. Another participant mentioned the representation of the family: “*I found the part about Ana with her son childish*” (P8). The stories were kept simple and dialogic, respecting the treatment process.

The almanac was considered relevant by most, with the exception of one participant who already had prior knowledge. The others reported that the material sparked interest, clarified doubts and expanded their information.

*It cleared up my doubts regarding social rights, there were a lot of things there that I didn’t know, even though I’m already finishing the whole process.* (P1)

[As] *to the issue of the cuticle itself, I removed it myself, I saw there that it’s not good to do that during chemotherapy.* (P6)

During the assessment interview, participants reported that reading the almanac and playing games improved their knowledge, as reported below:

*It cleared up a lot of my doubts regarding social rights.* (P1)

*I didn’t know much about the step-by-step process of radiotherapy; the explanation helped me a lot.* (P7)

## DISCUSSION

Educational technologies are important tools in health education, facilitating understanding and communication between health teams and patients; reinforcing and complementing verbalized guidelines; aiming to increase individuals’ knowledge about the disease and treatment; and, consequently, increasing adherence to self-care^(9-22,25,26)^.

The almanac was the type of educational technology selected by the participants of this research. This type of technology is a way of compiling knowledge with humorous, scientific and literary information without the need to go into depth about content. Therefore, it aims to teach, entertain and interact with readers through different approaches such as games, recipes, comic books, among others^(10)^.

Almanacs have been increasingly used in health education, addressing different topics and populations, especially in nursing. Stories and games help readers learn new information and reinforce their knowledge without realizing they are being instructed, allowing them to reflect on their attitudes and practices^(11,12)^.

Approximately 94% of participants in this survey had some knowledge about breast cancer and had received guidance from healthcare professionals; however, they still reported having many questions about different topics that they requested to compose the almanac.

Although people claim to have knowledge about breast cancer, studies conducted in different populations around the world reveal that they face difficulties in identifying risk factors and prevention strategies, in addition to lacking specific information about treatment. This gap in knowledge highlights the need for more effective educational actions to promote awareness and understanding about this disease^(7,27,28)^.

An international meta-analysis of women’s knowledge about breast cancer found that 84% of women knew about breast cancer. However, only 51% and 40% of women were aware of the symptoms and risk factors of breast cancer, respectively. The most well-known sign of breast cancer was a breast lump (71%), and a family history of breast cancer was the most well-known risk factor (61%)^(7)^.

In Jordan, approximately 76% of participants knew that breast cancer is the most common cancer among women. Furthermore, approximately 53.7% demonstrated an intermediate level of knowledge about risk factors associated with the disease, while 44% had a good to excellent level of knowledge about the signs and symptoms of breast cancer^(27)^.

A Brazilian study shows that 70% of people know the risk factors for breast cancer, while people with the lowest level of knowledge are those who have low education, do not work in the health sector, are male and have not had close contact with people with cancer^(28)^.

It is essential to improve health education about breast cancer at all stages of life in order to develop behaviors that promote health and allow early detection and control of risk factors associated with the development of cancer. To this end, healthcare professionals must have the knowledge, skills and competencies necessary to transmit relevant and up-to-date information on the subject^(29)^.

Thus, nursing professionals who work in healthcare services working in the prevention, diagnosis and treatment of breast cancer work in health education, developing skills to identify learning strategies that best suit the intended community. Nurses, through health education, provide knowledge, offering information that instructs individuals, the autonomy to make conscious choices, decisions about their treatment^(8)^.

Receiving information helps breast cancer patients effectively manage their health condition by improving their knowledge, encouraging proactive actions, monitoring their progress, managing symptoms, and promoting self-care^(8)^.

In addition, women with breast cancer report that their care and support needs include difficulties in performing household tasks, limitations in performing self-care activities, as well as the need for attention from healthcare professionals, education, and lifestyle advice^(30)^.

Healthcare professionals play an essential role in health education actions, promoting changes in thinking, improving self-care and encouraging the autonomy of cancer patients^(9,31)^.

Nursing interventions performed regularly during home visits made it possible to create positive changes in lymphedema prevention behavior and improve upper extremity functions, reduce side effects, alleviate symptoms in the operated arm and breast, improve quality of life, increase self-efficacy, and decrease lymphedema frequency and costs^(32)^.

In this context, the use of this almanac as an educational tool is important for healthcare professionals who are looking for reliable material adapted to the target population, since this type of material contributes significantly to promoting individual care and improving quality of life^(6,11,12)^.

### Study limitations

The technology assessment was carried out by a small number of participants undergoing treatment in just one health institution, which limits the universalization of the findings to the general population.

### Contributions to nursing and health

The almanac made it possible to translate dense and technical knowledge into a more fun, easy and simple language, facilitating the learning process for women with breast cancer.

## FINAL CONSIDERATIONS

This study presented the development and assessment of an educational technology aimed at women with breast cancer entitled “Almanac for Women with Breast Cancer”. This almanac is intended for all women diagnosed with this condition and their support networks. The work is a printed educational technology and is a self-explanatory resource, facilitating learning for both patients and healthcare professionals involved in their care.

The content covered was selected based on the needs identified during the interviews with the study participants. They considered the almanac to be a relevant, enlightening and effective tool in promoting knowledge and self-care.

In view of these results, it is recommended that the “Almanac for Women with Breast Cancer” be made widely available in healthcare services. This will contribute to the work of nurses in the health education process and also to the empowerment of women, informing and promoting self-care actions throughout treatment.

## Data Availability

Not applicable.

## References

[1] Ferlay J (2022). Global Cancer Observatory: cancer today.

[2] Instituto Nacional de Câncer José Alencar Gomes da Silva (Inca) (2023). Estimativas 2022.

[3] Shao J, Rodrigues M, Corter A, Baxter NN. (2019). Multidisciplinary care of breast cancer patients: a scoping review of multidisciplinary styles, processes, and outcomes. Curr Oncol.

[4] Barrios CH. (2022). Global challenges in breast cancer detection and treatment. Breast.

[5] Gutiérrez MG, Rosa AS. (2021). Detecção precoce do câncer de mama em Unidades Básicas de Saúde. Acta Paul Enferm.

[6] Lin L, Koh WL, Huang Q, Lee JK. (2021). Breast cancer information behaviours and needs among Singapore women: a qualitative study. Asian Pac J Cancer Prev.

[7] Wang Y-J, Wang F, Yu L-X, Xiang Y-J, Zhou F, Huang S-Y (2022). Worldwide review with meta-analysis of women’s awareness about breast câncer. Patient Educ Couns.

[8] An HJ, Kang SJ, Choi GE. (2023). Technology-based self-management interventions for women with breast cancer: a systematic review. Korean J Women Health Nurs.

[9] Ortega JMC, García RDE, Mares BH, Ortega JM. (2021). Educational interventions on breast cancer in men and women: a necessity in primary healthcare. Ecancermedicalscience.

[10] Silva BC, Primo CC, Almeida MVS, Cabral EI, Sant’Anna HC, Lima EFA. (2021). Pregnant women’s contribution in the construction and evaluation of an educational technology: the “Comics for Pregnant Women”. Rev Bras Enferm.

[11] Silva MY, Partelli ANM, Oliveira JD, Lopes MSV, Moreira MRL, Martins AKL. (2022). Almanac for preventing the use of alcohol and other drugs among adolescents: construction and validity. Rev Bras Enferm.

[12] Adriani PA, Hino P, Taminato M, Fernandes H. (2023). Construction of educational technology on non-violent communication between health professionals: an experience report. Rev Bras Enferm.

[13] Trentini M, Paim L, Silva DGV, Peres MAA. (2021). Pesquisa convergente assistencial e sua qualificação como investigação científica. Rev Bras Enferm.

[14] Souza VRS, Marziale MHP, Silva GTR, Nascimento PL. (2021). Tradução e validação para língua portuguesa e avaliação do guia COREQ. Act Paul Enferm.

[15] Bardin L. (2016). Análise de conteúdo.

[16] Presidência da República (BR) (2018). Lei nº 13.770 de 19 de dezembro de 2018. Dispões sobre cirurgia plástica reconstrutiva da mama em casos de mutilação decorrente de tratamento de câncer.

[17] Presidência da República (BR) (2012). Lei nº 12.732 de 22 de novembro de 2012. Dispõe sobre o primeiro tratamento de paciente com neoplasia maligna comprovada e estabelece prazo para seu início.

[18] Instituto Nacional de Câncer José Alencar Gomes da Silva (Inca) (2020). Direitos sociais da pessoa com câncer.

[19] Torres MJ. (2021). Mulheres mastectomizadas: cartilha de reabilitação.

[20] Silveira FM, Wysocki AD, Mendez RDR, Pena SB, Santos EM, Malaguti-Toffano S (2021). Impacto do tratamento quimioterápico na qualidade de vida de pacientes oncológicos. Acta Paul Enferm.

[21] AC Camargo Câncer Center (2019). A reabilitação de pacientes com câncer de mama.

[22] Paxman (2023). Pioneers in scalp cooling. Cuidados com o cabelo.

[23] Instituto Nacional de Câncer José Alencar Gomes da Silva (Inca) (2012). Cuidados após a cirurgia da mama com esvaziamento axilar: orientações aos pacientes.

[24] Inca (2023). Tratamento do câncer de mama.

[25] Leite SS, Áfio ACE, Carvalho LV, Silva JM, Almeida PC, Pagliuca LMF. (2018). Construção e validação de instrumento de validação de conteúdo educativo em saúde. Rev Bras Enferm.

[26] Sugisaka ACA, Andrzejevski VMS, Rotta I. (2020). Validação de materiais educativos para orientação de pacientes em tratamento de câncer de mama com hormonioterapia. Rev Bras Cancerol.

[27] Santibáñez-Ramírez M, Símbala-Delgado A, Valenzuela-Núñez N, Morales-Ojeda I, Gelaber-Santané R. (2019). Conocimiento del cáncer de mama en estudiantes de enfermería. Cien Enferm.

[28] Boaventura LF, Cima BP, Lindenau JD. (2022). How much do you know about breast cancer? assessing the level of knowledge of the Brazilian Population. Rev Bras Cancerol.

[29] Noman S, Shahar H, Abdul Rahman H, Ismail S. (2020). Effectiveness of an educational intervention of breast cancer screening practices uptake, knowledge, and beliefs among Yemeni female school teachers in Klang Valley, Malaysia: a stud protocol for a cluster-randomized controlled trial. Int J Environ Res Public Health.

[30] Donmez AA, Alici NK, Borman P. (2021). Lived experiences for supportive care needs of women with breast cancer-related lymphedema: a phenomenological study. Clin Nurs Res.

[31] Lourenço SC, Silva LCP, Laviola GM, Salles D, Lopes JL, Waitzberg AFL (2020). Entendendo o câncer de mama: educação em saúde. Enferm Foco.

[32] Cal A, Bahar Z, Gorken I. (2020). Effects of Health Belief Model based nursing interventions offered at home visits on lymphedema prevention in women with breast cancer: A randomised controlled trial. J Clin Nurs.

