# Supplementary material for: Construction and assessment of the “Almanac for Women with Breast Cancer”: convergent care research
Source: Rev Bras Enferm. 2025 Dec 12;78(Suppl 4):e20250031. doi: 10.1590/0034-7167-2025-0031 (PMC12707761; doi:10.1590/0034-7167-2025-0031)
Supplement: Translation

# Translation: Construção e avaliação do “Almanaque para a Mulher com Câncer de Mama”: pesquisa convergente assistencial

**DOI:** 10.1590/0034-7167-2025-0031pt

**Authors:** Karolina Rosa Teófilo, Denise Antunes de Azambuja Zocche, Ana Paula Carmona, Bruno Henrique Fiorin, Marcia Valéria de Souza Almeida, Cândida Caniçali Primo, Eliane de Fatima Almeida Lima

**Affiliations:** I Universidade Federal do Espírito Santo. Vitória, Espírito Santo, Brasil; II Universidade do Estado de Santa Catarina. Florianópolis, Santa Catarina, Brasil; III Escola Superior de Enfermagem de Lisboa. Lisboa, Portugal

**Keywords:** Neoplasias da Mama, Educação em Saúde, Tecnologia Educacional, Cuidados de Enfermagem, Mastectomia.

## Abstract

**Objetivos:**

desenvolver um almanaque impresso para mulheres com câncer de mama.

**Métodos:**

pesquisa convergente assistencial de abordagem qualitativa, realizada em serviço especializado em oncologia em Vitória, Espírito Santo, no período de agosto de 2023 a agosto de 2024. O almanaque foi construído baseado nas dúvidas das mulheres em tratamento quimioterápico. Após sua elaboração, as participantes usaram e avaliaram a tecnologia. Considerou-se como adequado o índice de concordância de 80%.

**Resultados:**

o almanaque possui 47 páginas, com histórias em quadrinhos e jogos como caça-palavras, sete erros, labirinto e outros. As participantes avaliaram a tecnologia educacional como relevante, esclarecedora e capaz de incentivar novos hábitos, além de agregar conhecimento. Todos os itens tiveram concordância acima de 80%.

**Conclusões:**

o almanaque consiste em uma tecnologia educacional inovadora que aborda fatores de risco, tratamentos, autocuidado e direitos sociais da pessoa com câncer de forma lúdica e com linguagem de fácil entendimento.

## INTRODUÇÃO

O câncer de mama continua sendo o câncer mais frequente e a principal causa de morte entre mulheres no mundo. Em 2022, segundo as estimativas do *Global Cancer Observatory*, dos 19,9 milhões de casos novos de câncer diagnosticados no mundo, 23,8% foram relacionados às neoplasias da mama. No Brasil, estima-se que, no triênio 2023-2025, ocorrerão 74 mil casos novos da doença, sendo 66,54 casos/100 mil mulheres. Destaca-se que o câncer de mama é a primeira causa de morte, com 16,47 óbitos/100 mil mulheres, no ano de 2020^(1,2)^.

As diferentes modalidades de tratamento para o câncer de mama expõem as mulheres a vários efeitos indesejáveis que devem ser avaliados pela equipe multiprofissional para o planejamento terapêutico que vise oferecer informações em saúde, conforto e promoção do autocuidado^(3,4)^.

As ações de educação em saúde constituem um dos pilares fundamentais para promoção e prevenção da saúde, ampliando o letramento em saúde das pessoas. Os materiais educacionais sobre câncer de mama devem fornecer informações claras e precisas para os pacientes^(5,6)^.

Muitas mulheres em todo o mundo ainda possuem um conhecimento limitado sobre os sintomas e fatores de risco do câncer de mama. Diante disso, é fundamental que os profissionais de saúde aprimorem as ações de educação em saúde voltadas para prevenção desse tipo de câncer, promovendo a conscientização sobre sua importância^(7)^. As tecnologias educacionais auxiliam nesse processo, pois podem ampliar o conhecimento das mulheres diagnosticadas com a doença e melhorar sua qualidade de vida, permitindo à paciente assumir seu protagonismo no tratamento^(8)^.

Nesse contexto, diferentes recursos tecnológicos e didáticos podem ser utilizados para realizar as intervenções educativas sobre câncer de mama, que são estratégias eficazes e de baixo custo a serem direcionadas a homens e mulheres, em todas as fases da vida^(9)^.

Entre as diversas tecnologias educacionais, os almanaques se destacam por serem informativos, humorísticos, lúdicos e dinâmicos. Eles facilitam a transição da cultura oral para a escrita, apresentando conteúdos de forma recreativa e sistematizada. Além disso, utilizam uma linguagem simples e de fácil compreensão, tornando a informação mais acessível para todos^(10-12)^.

Assim, considerando que o tratamento da doença, seja ele sistêmico, como as quimioterapias, ou local, como a radioterapia e as cirurgias, pode causar efeitos colaterais psicológicos, emocionais, sociais e físicos, as diferentes modalidades de tratamento devem ser abordadas pela equipe de saúde, a fim de reduzir as dúvidas e os medos das mulheres diagnosticadas^(7)^. Deste modo, desenvolveu-se estudo para a construção de um material educativo para as mulheres diagnosticadas com câncer de mama, a fim de retirar suas dúvidas sobre o tratamento, bem como de apoio para uso dos profissionais de saúde durante as orientações verbais. Nesse sentido, a participação do público-alvo nessa construção foi fundamental para atender às suas reais necessidades.

## OBJETIVOS

Desenvolver um almanaque impresso para mulheres com câncer de mama.

## MÉTODOS

### Aspectos éticos

O projeto de pesquisa foi aprovado pelo Comitê de Ética e Pesquisa com Seres Humanos, sob Certificado de Apresentação para Apreciação Ética nº 70587623.6.0000.5060.

As participantes foram informadas sobre o objetivo e etapas de desenvolvimento da pesquisa. Após leitura do Termo de Consentimento Livre e Esclarecido, as participantes assinaram e receberam uma cópia do termo. As entrevistas foram realizadas em local reservado, e o anonimato dos participantes foi preservado mediante a utilização da letra “P” para identificar cada entrevistada. As participantes foram informadas de que poderiam sair da pesquisa a qualquer momento e que não haveria implicações na continuidade do seu cuidado.

### Referencial teórico-metodológico

O estudo utilizou o método da pesquisa convergente assistencial (PCA), que consiste na articulação entre ações de pesquisa e prática assistencial, permitindo a aquisição de informações em ambos os processos^(13)^.

### Cenário do estudo

O estudo foi realizado em um serviço especializado em oncologia do município de Vitória, Espírito Santo, no período de agosto de 2023 a agosto de 2024.

A pesquisa seguiu as etapas descritas pelo modelo metodológico da PCA, que consiste na concepção, instrumentação, perscrutação e análise^(13)^.

Na fase de concepção, foi delimitado o tema das questões de pesquisa a partir da prática assistencial da pesquisadora, que assumiu um papel proativo, buscando a convergência entre ações de pesquisa e de assistência. Ainda nessa fase, foi realizada revisão bibliográfica narrativa sobre a temática a fim de aproximar o conhecimento da pesquisadora com as evidências encontradas na literatura. Com base nesses resultados, a proposta de pesquisa foi apresentada à equipe de enfermagem da instituição antes de iniciar a fase de coleta de dados, a fim de envolvê-los no estudo para que os problemas que surgissem a partir dos dados obtidos pudessem ser compartilhados com a finalidade de promover a discussão e resolução da problemática.

Na etapa de instrumentação, foram definidas as questões metodológicas, como o cenário da pesquisa, a caracterização das participantes e a técnica de coleta de dados.

As participantes foram mulheres com câncer de mama em quimioterapia, selecionadas por conveniência em função do seu envolvimento com o assunto, conforme o comparecimento no local para tratamento.

Foram incluídas mulheres diagnosticadas com câncer de mama em tratamento e alfabetizadas. Foram excluídas mulheres submetidas à cirurgia curativa como único tratamento (este procedimento é realizado em serviço externo) e as que apresentaram história pessoal de doença psiquiátrica ou cognitiva.

A coleta de dados ocorreu por meio de entrevistas individuais semiestruturadas realizadas durante o tratamento infusional da participante. No primeiro momento, foi apresentada a pesquisa, lido o Termo de Consentimento Livre e Esclarecido e, após ser assinado, foi realizada a entrevista. Não houve recusas. Para nortear a pesquisa, foi utilizado o instrumento *COnsolidated criteria for REporting Qualitative research* ^(14)^.

As 16 entrevistas aconteceram de 08 de outubro de 2023 a 06 de fevereiro de 2024; foram gravadas em MP3 e duraram aproximadamente 30 minutos. Encerraram-se as entrevistas quando ocorreu a saturação teórica dos dados, não surgindo conteúdo novo.

Durante a entrevista, foi utilizado um formulário estruturado pela autora para identificar os dados sociodemográficos e econômicos, e o tipo de tecnologia educacional de escolha da participante (almanaque, livro-jogo, gibi e caça-palavras). Ainda na entrevista, foi perguntado sobre quais orientações as participantes receberam dos profissionais de saúde após o diagnóstico, seus conhecimentos acerca do autocuidado e dos direitos sociais da pessoa com câncer, sobre quais tratamentos realizaram, e se foram orientadas quanto aos possíveis efeitos colaterais.

As gravações das entrevistas foram transcritas na íntegra pela autora e lidas exaustivamente. Para a análise de conteúdo categorial, conforme descrita por Bardin, seguiram-se as fases de pré-análise, exploração do material, e tratamento dos resultados, inferência e interpretação^(15)^.

Na fase de perscrutação, foi desenvolvida a tecnologia educacional no formato de almanaque (tipo escolhido pelas participantes), contemplando conteúdos sugeridos a partir das dúvidas apresentadas por elas ou temas que elas julgaram importantes para compor o material.

Para a fundamentação teórica, foi realizada a revisão bibliográfica narrativa sobre os principais temas identificados nas entrevistas. Buscaram-se conteúdos nos manuais do Ministério da Saúde, cartilhas relacionadas ao câncer de mama, artigos científicos publicados na Biblioteca Virtual em Saúde, nas bases de dados *Medical Literature Analysis and Retrieval System Online*, Literatura Latino-Americana e do Caribe em Ciências da Saúde e Base de Dados em Enfermagem^(16-24)^. Para a seleção de jogos educacionais, procedeu-se ao levantamento em revistas de passatempos, cartilhas e almanaques existentes.

A fase de análise e interpretação dos dados subsidiou o processo de elaboração do almanaque seguindo as fases de organização do conteúdo fundamentado em pesquisas científicas, elaboração do modelo piloto, ilustração e diagramação do almanaque. Nesse sentido, o protótipo do almanaque foi elaborado a partir de fotos feitas pela pesquisadora, representando os diversos cenários de tratamento da mulher com câncer de mama, a fim de expressar a ideia proposta pela tecnologia educacional. O conteúdo do protótipo foi corrigido quanto à ilustração, diagramação e linguagem, com a colaboração de uma equipe constituída por *designer*, profissional de letras e de saúde.

Na etapa de avaliação, as mesmas participantes receberam o almanaque, e após uma semana de uso do material, foram entrevistadas novamente. As entrevistas aconteceram de julho a agosto de 2024. Na entrevista, foi aplicado o formulário adaptado e baseado no instrumento de validação de conteúdo educativo em saúde com objetivos, estrutura/apresentação e relevância do material^(25)^, e poderiam solicitar esclarecimento caso julgassem necessário. Elas assinalaram com um “x”, conforme seus julgamentos, as seguintes variáveis dicotômicas: 1 - concordo; e 2 - não concordo. Os itens que atingiram concordância mínima de 80% não foram alterados, por serem considerados adequados. Durante a entrevista, as participantes foram questionadas sobre como foi para elas lerem e usar o almanaque, quais suas percepções acerca do material, os pontos positivos e o que poderia ser melhorado.

## RESULTADOS

Participaram da pesquisa 16 mulheres com diagnóstico de câncer de mama, em tratamento medicamentoso endovenoso, com média de idade de 46,9 anos. Destas, 62,5% possuíam ensino superior completo; 93,75% relataram emprego fixo; e 81,2% eram casadas. Em relação ao número de filhos, 75% das mulheres tinham entre um ou dois filhos e residiam em municípios da grande Vitória, Espírito Santo, Brasil.

A tecnologia educacional desenvolvida intitulada “Almanaque para a Mulher com Câncer de Mama” consiste em um material impresso com 47 páginas coloridas, produzidas em papel couchê, com dimensões de 21 cm de altura por 15 cm de largura. O conteúdo é apresentado por meio de histórias e jogos protagonizados pelos personagens Ana (paciente), Zeca (marido), Rafa (filho), vovó Lena e enfermeira Flora. Parte desse material é descrito ao longo deste artigo.

A capa do almanaque é atrativa e busca transmitir acessibilidade, parceria e segurança entre a mulher e o profissional de saúde (Figura 1). Sua proposta é reduzir o distanciamento relatado pelas participantes, causado por orientações fragmentadas e incompletas durante o tratamento.

**Figura 1:** Capa do almanaque e apresentação dos personagens, Vitória, Espírito Santo, Brasil, 2024

Foram elaboradas oito histórias em quadrinhos com temas escolhidos pelas participantes, como mastectomia, pós-operatório, fatores de risco, direitos sociais, quimioterapia, radioterapia, lazer e conclusão do tratamento. Para estimular o aprendizado, o almanaque inclui dicas da enfermeira, receitas e 11 passatempos, como caça-palavras e cruzadinhas. Também apresenta um calendário para planejamento do tratamento.

Sobre o conteúdo relacionado às cirurgias (mastectomia e quadrantectomia), destacam-se os cuidados com o dreno, pois, das 13 participantes que realizaram algum procedimento cirúrgico, nove utilizaram o dispositivo no pós-operatório e apenas seis foram orientadas, porém de forma inadequada, com informações incompletas e sem embasamento científico, como demonstrado em:

*Em relação ao dreno, acho que o cuidado era passar um remedinho, a rifocina.* [...] *tipo merthiolate, eu usei muito.* (P8)

*Eles falaram para o meu marido: “Quando encher o reservatório do dreno, você vai lá no vaso, abre isso aqui e vira e fecha”. Só que, um dia, eu estava dormindo e o reservatório soltou, eu falei com meu marido: “Lava o trem* [dreno] *agora e coloca de novo”.* (P16)

Para orientar as mulheres sobre a mastectomia e os cuidados pós-procedimento, foram elaborados jogos e dicas sobre o assunto (Figura 2).

**Figura 2:** Informações sobre mastectomia e cuidados após a cirurgia no almanaque, Vitória, Espírito Santo, Brasil, 2024

As participantes também trouxeram questões relacionadas aos fatores de riscos para o desenvolvimento do câncer de mama, recidivas e o tipo de câncer HER2+, conforme exposto nas falas:

*Eu não lembro os fatores de riscos para o desenvolvimento do câncer de mama.* (P12)

*Na realidade, eu sempre tenho um pouco de dúvidas.* [...] *eu estou em uma recidiva, aí fico pensando no “que posso fazer para isso não acontecer novamente?”. Será que eu errei? Será que alguma coisa eu poderia ter feito? Por que isso aconteceu?* (P14)

O tratamento quimioterápico e a radioterapia foram apresentados por meio de histórias em quadrinhos contextualizando o tratamento, bem como os cuidados para prevenção de reações adversas e uso da crioterapia. Também foram elaborados jogos e dicas sobre a temática, a fim de traduzir o conhecimento de forma simples, lúdica e de fácil compreensão (Figura 3).

**Figura 3:** Informações sobre o desenvolvimento do câncer, tratamentos e cuidados durante a radioterapia, Vitória, Espírito Santo, Brasil, 2024

O almanaque incluiu sugestões de exercícios físicos para a prevenção do linfedema e alguns direitos sociais garantidos por lei para pessoas com diagnóstico de câncer, como o auxílio-doença, isenção de imposto de renda, aposentadoria por invalidez e outros (Figura 4).

**Figura 4:** Páginas do almanaque com informações sobre exercícios para prevenção do linfedema e quadros descrevendo alguns direitos sociais da pessoa com câncer, Vitória, Espírito Santo, Brasil, 2024

*Não sei o que é linfedema.* [...] *o médico apenas me falou para eu não fazer força com esse braço do lado que fiz a cirurgia.* (P8)

*No momento, não sei nenhum direito social que tenho por estar fazendo tratamento, mas gostaria de saber.* (P10)

O almanaque finaliza com a história “Ana em: finalizando o tratamento”, em que Ana e Zeca retornam em consulta com a enfermeira Flora, que orienta sobre o acompanhamento após o término do tratamento.

O gabarito com as respostas dos jogos foi inserido na última página do “Almanaque para a Mulher com Câncer de Mama”.

As informações seguiram uma ordem cronológica que pode divergir, visto que o tratamento é definido de acordo com as características de cada doença e a condição clínica de cada pessoa.

Na fase de avaliação do almanaque, houve a perda de seis participantes devido a óbito, dificuldade de comunicação para a entrega da tecnologia educacional e para agendamento da entrevista, e progressão de doença, incapacitando a leitura. Deste modo, dez mulheres responderam ao formulário de avaliação.

Na avaliação da tecnologia, todos os itens alcançaram concordância acima de 80%; portanto, foram considerados adequados.

A maioria das participantes (90%) relatou que o conteúdo é informativo, incentiva novos hábitos e aborda questões importantes em que a maioria das mulheres possui dúvidas.

*Tudo que está ali, para mim, está ótimo, me tirou muitas dúvidas, principalmente a questão dos direitos sociais.* [...] *muita coisa ali que eu não sabia.* (P1)

*Incentiva novos hábitos tanto alimentares quanto à importância da atividade física.* (P3)

*Então, quem me dera se eu tivesse um almanaque desse quando eu fui diagnosticada. Achei que ele aborda as informações de forma muito clara.* (P7)

Por outro lado, 10% discordaram de que o almanaque atendeu a todos os temas propostos por não tratar com profundidade os tipos de neoplasias da mama como HER2 positivo ou negativo, triplo negativo etc. No entanto, esses itens não foram incluídos porque essas informações devem ser dadas pelo profissional de saúde com o alinhamento do protocolo que será seguido, sendo o almanaque uma tecnologia de apoio para o processo de educação em saúde.

Em relação à estrutura/apresentação, a maioria das participantes julgou o material como objetivo e esclarecedor, além de ser importante a inclusão da rede de apoio:

*O material é leve, descomplicado e informativo. Aborda questões importantes que a grande maioria dos pacientes possuem dúvidas.* (P2)

*Eu gostei, gostei muito, eu achei ótimo, muito explicativo.* [...] *achei criativos e divertidos os joguinhos.* (P9)

*Achei interessante a inclusão da família durante o tratamento.* [...] *as cores e o material da impressão são favoráveis à leitura.* (P13)

Uma participante achou a linguagem infantilizada nas histórias em quadrinhos e sugeriu trocar “medir pressão arterial” por “aferir pressão arterial” (página 04). Para ampliar o vocabulário, ambos os termos foram mantidos, com um entre parênteses. Outra participante mencionou a representação da família: *“A parte da Ana com o filho achei infantilizada”* (P8). As histórias mantiveram-se simples e dialogais, respeitando o processo do tratamento.

O almanaque foi considerado relevante pela maioria, com exceção de uma participante que já possuía conhecimento prévio. As demais relataram que o material despertou interesse, esclareceu dúvidas e ampliou suas informações.

*Tirou minha dúvida em relação aos direitos sociais, muita coisa ali que eu não sabia, por mais que já esteja finalizando o processo todo.* (P1)

E*m questão da cuticulagem mesmo, eu mesma tirava, eu vi ali que não é legal fazer isso durante a quimioterapia.* (P6)

Durante a entrevista de avaliação, as participantes relataram que a leitura do almanaque e os jogos melhoraram o conhecimento delas, conforme relatos a seguir:

*Tirou muito da minha dúvida em relação aos direitos sociais.* (P1)

*Eu não conhecia muito o passo a passo da radioterapia; me ajudou bastante a explicação.* (P7)

## DISCUSSÃO

Tecnologias educacionais são ferramentas importantes na educação em saúde, facilitando a compreensão e a comunicação entre a equipe de saúde e o paciente; reforçando e complementando as orientações verbalizadas; visando o aumento do conhecimento do indivíduo sobre a doença e o tratamento; e, consequentemente, aumentando a adesão ao autocuidado^(9-22,25,26)^.

O almanaque foi o tipo de tecnologia educacional selecionado pelas participantes desta pesquisa. Esse tipo de tecnologia é uma forma de compilar o conhecimento com informações humorísticas, científicas e literárias sem a necessidade de aprofundar o conteúdo. Portanto, tem o objetivo de ensinar, distrair e interagir com o leitor por meio de diferentes abordagens como os jogos, as receitas, as histórias em quadrinhos, entre outras^(10)^.

Os almanaques têm sido cada vez mais utilizados na educação em saúde, abordando diferentes temáticas e populações, em especial na área da enfermagem. As histórias e jogos favorecem que o leitor aprenda novas informações e reforce seu conhecimento sem perceber que está sendo instruído, permitindo refletir sobre suas atitudes e práticas^(11,12)^.

Aproximadamente 94% das participantes desta pesquisa possuíam algum conhecimento sobre câncer de mama e tinham sido orientadas por profissionais de saúde; no entanto, ainda relataram apresentar muitas dúvidas sobre diferentes temas que solicitaram compor o almanaque.

Embora as pessoas afirmem ter conhecimento sobre câncer de mama, estudos realizados em diversas populações ao redor do mundo revelam que elas enfrentam dificuldades em identificar fatores de risco e estratégias de prevenção, além de carecer de informações específicas sobre o tratamento. Essa lacuna no conhecimento evidencia a necessidade de ações educacionais mais efetivas para promover a conscientização e compreensão sobre essa doença^(7,27,28)^.

Revisão internacional com meta-análise acerca do conhecimento das mulheres sobre o câncer de mama apontou que 84% das mulheres sabiam sobre o câncer de mama. No entanto, apenas 51% e 40% das mulheres estavam cientes dos sintomas e fatores de risco do câncer de mama, respectivamente. O sinal mais conhecido do câncer de mama foi o nódulo mamário (71%), e o histórico familiar de câncer de mama foi o fator de risco mais conhecido (61%)^(7)^.

Na Jordânia, aproximadamente 76% dos participantes sabiam que o câncer de mama é o mais comum entre mulheres. Além disso, cerca de 53,7% demonstraram nível intermediário de conhecimento sobre fatores de risco associados à doença, enquanto 44% apresentavam nível de conhecimento bom a excelente sobre sinais e sintomas do câncer de mama^(27)^.

Estudo brasileiro aponta que 70% das pessoas conhecem os fatores de risco para câncer de mama, enquanto as pessoas com menor nível de conhecimento são as que possuem baixa escolaridade, não trabalham na área da saúde, são do sexo masculino e não tiveram contato próximo com pessoas com câncer^(28)^.

É essencial aprimorar a educação em saúde sobre câncer de mama em todas as fases da vida, a fim de desenvolver comportamentos que promovam a saúde e permitam a detecção e controle precoce dos fatores de riscos associados ao desenvolvimento do câncer. Para isso, os profissionais de saúde devem possuir conhecimentos, habilidades e competências necessárias para transmitir informação relevante e atualizada sobre o tema^(29)^.

Nesse sentido, o profissional da enfermagem que está inserido nos serviços de saúde atuando na prevenção, diagnóstico e tratamento do câncer de mama atua na educação em saúde desenvolvendo habilidades para identificar estratégias de aprendizagem que mais se adequa à comunidade que se destina. O enfermeiro, por meio da educação em saúde, proporciona saberes, oferecendo informações que instruem o indivíduo, a autonomia de escolherem de forma consciente, decisões sobre seu tratamento^(8)^.

Receber informação ajuda as pacientes com câncer de mama a gerenciar eficazmente sua condição de saúde, melhorando seu conhecimento, encorajando ações proativas, monitorando seu progresso, gerenciando sintomas e promovendo o autocuidado^(8)^.

Além disso, mulheres com câncer de mama relatam que, entre suas necessidades de cuidados e suporte, estão as dificuldades em realizar tarefas domésticas, limitações em realizar atividades de autocuidado, bem como necessitam de atenção dos profissionais de saúde, educação e conselhos sobre estilo de vida^(30)^.

Os profissionais da saúde desempenham um papel essencial nas ações de educação em saúde, promovendo mudanças de pensamento, melhoria no autocuidado e incentivando a autonomia dos pacientes oncológicos^(9,31)^.

Intervenções de enfermagem realizadas regularmente durante visitas domiciliares possibilitaram criar mudanças positivas no comportamento de prevenção do linfedema e melhorar as funções das extremidades superiores, reduzir efeitos colaterais, aliviar sintomas no braço e mama operados, melhorar a qualidade de vida, aumentar a autoeficácia, e diminuir a frequência de linfedema e custos^(32)^.

Nesse contexto, o uso deste almanaque como ferramenta educativa é importante para os profissionais da saúde que buscam um material confiável e adaptado à população a que se destina, já que este tipo de material contribui significativamente para a promoção de cuidados individuais e a melhoria da qualidade de vida^(6,11,12)^.

### Limitações do estudo

A avaliação da tecnologia foi realizada por um número reduzido de participantes em tratamento em apenas uma instituição de saúde, o que restringe a universalização dos achados para a população geral.

### Contribuições para as áreas da enfermagem e saúde

O almanaque possibilitou a tradução de conhecimentos densos e técnicos para uma linguagem mais divertida, fácil e simples, facilitando o processo de aprendizado das mulheres com câncer de mama.

## CONSIDERAÇÕES FINAIS

Este estudo apresentou o desenvolvimento e avaliação de tecnologia educacional voltada para mulheres com câncer de mama intitulada “Almanaque para a Mulher com Câncer de Mama”. O almanaque é destinado a todas as mulheres diagnosticadas com essa condição e suas redes de apoio. A obra é tecnologia educacional impressa e se configura recurso autoexplicativo, facilitando o aprendizado tanto para as pacientes quanto para os profissionais de saúde envolvidos em seu cuidado.

Os conteúdos abordados foram selecionados com base nas necessidades identificadas durante as entrevistas com as participantes do estudo. Elas consideraram o almanaque como uma ferramenta relevante, esclarecedora e eficaz na promoção do conhecimento e autocuidado.

Diante desses resultados, recomenda-se que o “Almanaque para a Mulher com Câncer de Mama” seja amplamente disponibilizado nos serviços de saúde. Isso contribuirá para a atuação dos enfermeiros no processo de educação em saúde e também para o empoderamento das mulheres, informando e promovendo ações de autocuidado ao longo do tratamento.

## Data Availability

Não se aplica.
